# *In vitro* study of gut microbiota-mediated response to *Salmonella* Typhimurium with a focus on dynamics and immune modulation

**DOI:** 10.1080/21505594.2026.2758464

**Published:** 2026-11-05

**Authors:** Dong Woo Kim, Saloni Singh, Hyeon Ji Je, Jun Hong Shin, Ui in Kim, Dong Young Lee, Eun Ju Yun, Seung Ho Shin, Ok Kyung Koo

**Affiliations:** a Department of Food Science & Technology, Chungnam National University, Daejeon, South Korea; b Department of Food and Nutrition, Gyeongsang National University, Jinju, South Korea; c Department of Biotechnology, The Catholic University of Korea, Bucheon, South Korea; d Department of Bio and Medical Bigdata (BK4 Program), Gyeongsang National University, Jinju, South Korea

**Keywords:** Gut microbiota, *Salmonella* Typhimurium, fecal fermentation model, resilience

## Abstract

*Salmonella enterica* serovar Typhimurium is a major foodborne pathogen that disrupts gut microbial balance and induces intestinal inflammation. Microbial analysis revealed that *S*. Typhimurium infection increased the abundance of short-chain fatty acid (SCFA)-producing bacteria, including *Bacteroides*, *Parabacteroides*, and members of the Clostridia group, while decreasing *Escherichia-Shigella* in an *in vitro* fecal fermentation model. Co‑culture experiments demonstrated that gut microbiota inhibited *S*. Typhimurium growth and host cell interactions while attenuating cytotoxicity and inflammatory responses (IL‑1β, IL‑6, and c‑Jun) in Caco-2 cells in a dose‑dependent manner. Interestingly, gut microbiota improved epithelial cell viability, indicating barrier‑protective and immunomodulatory functions. These findings demonstrate that human gut microbiota plays a crucial role in mitigating the pathogenicity of *S*. Typhimurium and maintaining intestinal homeostasis, supporting the potential of microbiota‑based strategies for infection control.

## Graphical abstract

**Figure:**
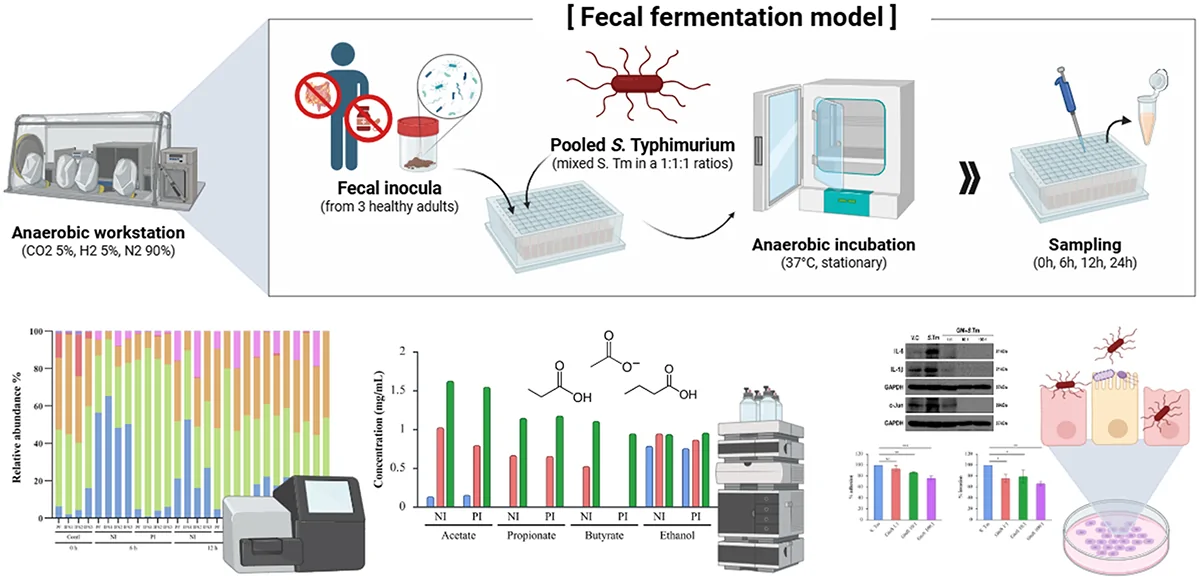

## Introduction

Gut microbiota is essential for human health, contributing to immune regulation, nutrient metabolism, and resistance against pathogenic colonization, thereby maintaining gastrointestinal homeostasis [1]. Its composition and function are shaped by host dietary habits and environmental factors, which influence its resilience and protective capacity [1]. One of the primary defense mechanisms of gut microbiota involves competitive exclusion of pathogens through nutrient competition, production of antimicrobial compounds such as bacteriocins and short-chain fatty acids (SCFAs), and occupation of epithelial adhesion sites [2,3]. Propionate produced by commensal *Bacteroides* spp. suppresses enteric pathogens by disrupting intracellular pH homeostasis, thereby extending their lag phase and reducing growth rates [4]. Butyrate reinforces tight junctions, promotes the differentiation of regulatory T cells, and suppresses pro-inflammatory pathways such as NF-κB signaling [3,5]. These barrier-protective and immunoregulatory functions of gut microbiota underscore its crucial role in host defense and resistance to enteric infections.

*Salmonella enterica* serovar Typhimurium is a highly virulent pathogenic bacterium that causes approximately 150 million cases and 60,000 deaths worldwide each year through the contamination of food or water [3,6]. Once ingested through contaminated food, the pathogen manipulates the gut environment to favor its expansion by inducing inflammation and reshaping the gut microbial composition [2]. The disruption of the intestinal epithelium not only decreases epithelial oxygen consumption but also triggers immune responses that generate reactive oxygen species, together creating a more oxygen-rich and thus aerobic intestinal environment [7]. This shift promotes facultative anaerobes, such as *Escherichia*, *Klebsiella*, and *Enterobacter* species, leading to dysbiosis characterized by the loss of a balanced microbial composition and function [8,9]. This dysbiosis impairs the colonization resistance provided by gut microbiota and enables *S*. Typhimurium to exploit nutrients and ecological niches vacated by beneficial microbes [7,9]. Following expansion in the intestinal lumen, *S*. Typhimurium adheres to and invades epithelial cells, compromising the epithelial barrier and eliciting heightened inflammatory responses through the activation of host immune pathways [10].

Animal studies are among the most commonly used approaches for studying pathogens and their pathogenesis [11]. Different mouse strains harbor distinct gut microbiota compositions and that even mice of the same strain can exhibit vendor-dependent differences, resulting in variable *S*. Typhimurium colonization resistance [12,13]. Moreover, host-specific immune responses and dietary behaviors in animal studies can influence how well findings translate from animal models to humans [11]. Significant differences exist when the microbial community of humans is compared to that of animal models [14]. In a mouse model, Bacillota (formerly Firmicutes) members are poorly established, leading to an enrichment of Bacteroidota (formerly Bacteroidetes), resulting in Bacillota‑to‑Bacteroidota ratios below 1.6 (approximately 38% Bacillota). In contrast, specific pathogen-free mice showed ratios greater than 6.8 (approximately 87% Bacillota), human microbiota-associated rats had ratios over 2.4 (approximately 71% Bacillota), and the human donor exhibited a ratio of 6.3 (approximately 86% Bacillota) [14]. Such differences can critically affect the interpretation of results. Therefore, conducting and comparing *in vivo* and *in vitro* studies of gut microbiota is important. These efforts are necessary to develop effective alternatives and to gain a deeper understanding of the synergistic interactions within the microbiome, thereby fully harnessing its potential during infections. The fecal fermentation model is an *in vitro* approach that employs human fecal samples as inocula, enabling the stable maintenance and reproduction of the human gut microbiota [15]. The model proposed by Li et al. (2019) was based on a 96-deep well plate format, allowing the efficient processing of multiple samples in small volumes [15]. In our previous study, this model was employed to investigate the interactions between the gut microbiota and *Listeria monocytogenes* [16].

This study aimed to investigate the dynamic interactions between human gut microbiota and *S*. Typhimurium during infection, using an established *in vitro* human fecal fermentation model [15]. Additionally, we examined how the presence of gut microbiota influences pathogenesis of *S*. Typhimurium, including adhesion, invasion, and cytotoxicity toward intestinal epithelial cells, as well as its impact on host immune responses. Ultimately, this research contributes to a broader understanding of host-microbe-pathogen interactions and supports the development of microbiota-driven approaches to enhance gut health and strengthen host resilience against pathogenic challenges.

## Materials and methods

### Bacterial strains and cell line

The *S*. Typhimurium strains DT104, U302, and ATCC 14028 used in this study were obtained from the strain collection of the Food Safety Laboratory, Chungnam National University (Daejeon, Republic of Korea). The strains were maintained in 15% glycerol in Brain Heart Infusion (BHI; Kisan Bio, Seoul, South Korea) broth at −80°C. BHI broth and agar were used for pre-enrichment, and Xylose Lysine Deoxycholate (XLD; Becton Dickinson, Franklin Lakes, NJ, USA) agar was used for selective enrichment. For the *S*. Typhimurium cocktail, each strain was cultured for 18 h and then mixed at a 1:1:1 ratio. A cocktail of three strains was used to minimize the effects of strain-specific characteristics and to evaluate the overall response of the gut microbiota to *S*. Typhimurium.

Caco-2 cells maintained in the Food and Enzyme Laboratory of Chungnam National University were used in this study. The cells, with passage numbers ranging from 17 to 22, were cultured in Dulbecco’s Modified Eagle’s Medium (DMEM, GenDEPOT, Katy, USA) supplemented with 10% (*v/v*) fetal bovine serum (FBS, GenDEPOT). The cells were maintained in a humidified incubator at 37°C with 5% CO_2_ and were used when they reached 90–95% confluence.

### Ethics and criteria for sample collection

Fecal samples (4–5 g) were obtained from three healthy adult volunteers following approval by the Institutional Review Board of Chungnam National University (CNU-IRB, Approval No. 202,209-BR-125–01). The study was conducted in accordance with the Declaration of Helsinki, and written informed consent was obtained from all participants prior to enrollment. Donors were recruited through posted advertisements within Chungnam National University and were eligible if they had not taken antibiotics, probiotics, or prebiotics within 1-month prior to sample collection [17]. Samples were self-collected using sterilized stool containers (SPL Life Sciences, Pocheon, South Korea) on 30 November 2023, and transported to the laboratory immediately after collection. For fecal fermentation experiments, samples were stored at 4°C and processed within 6 h.

### In vitro fecal fermentation

The *in vitro* fecal fermentation was conducted with minor modifications to the method described by Li et al. (2019) [15]. Fecal samples (2–3 g) were collected from three healthy volunteers (IFS1, IFS2, and IFS3) and each sample was suspended in 20 mL of sterile phosphate-buffered saline (PBS; Gibco BRL, Paisley, UK) containing 0.5 g/L L-cysteine (Junsei, Tokyo, Japan). The mixtures were vigorously vortexed to ensure complete homogenization and then filtered through four layers of sterile gauze to remove large debris. In addition to the three individual fecal samples, a pooled fecal sample (PF), prepared by mixing equal proportions of IFS1, IFS2, and IFS3, was included to provide a more representative composite fecal microbial community. All procedures involving gut microbiota were performed under strict anaerobic conditions (5% H_2_, 5% CO_2_, 90% N_2_) at 37°C in a Coy Anaerobic Vinyl Chamber (Coy Laboratory Products, MI, USA) to minimize oxygen exposure and preserve the native composition and metabolic activity of the gut microbiota.

The MiPro medium used for fecal fermentation was formulated according to Li et al. (2019) [15] and prepared following the composition described previously [16]. Approximately 7 log gene copies (GC)/mL of *S*. Typhimurium cocktail and gut microbiota in fecal slurry were inoculated into the medium. Fermentation was carried out in 96-deep well plates (Eppendorf, Hamburg, Germany) sealed with punctured silicone mats and incubated at 37°C. Samples were collected at 0, 6, 12, and 24 h. The survival of *S*. Typhimurium was quantified by plating aliquots of the fermentation samples onto XLD agar. The remaining samples were centrifuged at 8,000 rpm for 10 min, and the resulting supernatants were filtered using a 0.22 μm syringe filter (13 mm, polytetrafluoroethylene; Advantec, Tokyo, Japan). Both pellets and supernatants were stored at −80°C for subsequent analysis.

### Quantitative PCR and 16S rRNA amplicon sequencing

To quantify the bacterial population, total DNA was extracted from fermented samples using a NucleoSpin DNA Stool Kit (Macherey-Nagel, Düren, Germany). Quantitative PCR (qPCR) assays were conducted on a CFX Connect Real-Time PCR System (Bio-Rad, Hercules, CA, USA). Each 20 μL reaction mixture contained 10 μL of iQ SYBR Green Supermix (Bio-Rad), 0.5 μM of the forward (5′-GTGSTGCAYGGYYGTCGTCA-3′) and reverse (5′-ACGTCRTCCMCNCCTTCCTC-3′) primers, along with template DNA, as described by Fuller et al. (2007) [18]. A standard curve was constructed using serial dilutions (10^4^-10^9^ GC/ng) of pMG-AMP plasmids (Macrogen Co., Seoul, South Korea) harboring *Bifidobacterium miconisargentati* 16S rRNA gene (accession ID: NR_181805.1). Amplification was performed under the following thermal cycling conditions: an initial denaturation at 95°C for 3 min, followed by 40 cycles of denaturation at 95°C for 5 s and annealing/extension at 60°C for 30 s.

16S rRNA amplicon sequencing was conducted by Sanigen Co. Ltd. (Anyang, South Korea) and analyzed using the same workflow as described previously [16]. The sequencing data are available in the NCBI Sequence Read Archive (SRA) under BioProject accession number PRJNA1141279 (https://www.ncbi.nlm.nih.gov/sra/PRJNA1141279).

### SCFAs analysis

The concentrations of SCFAs in fermentation supernatants were quantified using a Nexera high-performance liquid chromatography (HPLC) system (Shimadzu, Kyoto, Japan) coupled with a RID-20A refractive index detector (Shimadzu) and an Aminex HPX-87 H analytical column (300 × 7.8 mm; Bio-Rad, Hercules, CA, USA). Each sample (20 µL) was injected into the column, and compounds were separated over a 25-min run under isocratic elution. The mobile phase was 0.008 N sulfuric acid (Sigma-Aldrich, St. Louis, MO, USA) delivered at a flow rate of 0.6 mL/min, while the column compartment was maintained at 35°C throughout analysis. Calibration curves were generated using authentic standards of acetate, propionate, butyrate, and ethanol (Sigma-Aldrich) to enable compound identification and quantification.

### Adhesion and invasion assay

Adhesion and invasion studies were performed according to previous studies [19]. Briefly, Caco-2 cells were seeded in 24-well plates at a density of 2 × 10^5^ cells/well, with the medium changed every 3 days. The cells were cultured in DMEM supplemented with 10% FBS (D10F). Following 10 days of proliferation, the cells were washed twice with PBS and then inoculated with five treatment groups in D10F: (1) *S*. Typhimurium cocktail alone at a multiplicity of infection 100 (MOI 100), (2)-(4) co-cultures of PF slurry and *S*. Typhimurium at gut microbiota-to-*S*. Typhimurium (GtoS) ratios of 1:1, 10:1, and 100:1, respectively. After 1 h incubation, the wells were washed five times with PBS. The Caco-2 cells were then treated with 0.1% Triton X-100 (Sigma-Aldrich) and plated onto XLD agar to quantify *S*. Typhimurium.

The invasion assay was performed using the same procedure as the adhesion assay, with the infection time modified from 1 h to 2 h. The cells were then incubated with 200 μg/mL gentamicin (Sigma-Aldrich) for 1 h to kill extracellular bacteria. After gentamicin treatment, the cells were lysed with 0.1% Triton X-100, and the concentration of *S*. Typhimurium was determined by plating onto XLD agar.

### Cytotoxicity assay

Cytotoxicity assays were performed using a CyQUANT™ LDH Cytotoxicity Assay (Invitrogen, Carlsbad, CA, USA) [20]. Caco-2 cells (2 × 10^4^ cells/well) were seeded and incubated for 15–16 h for their adherence on a 96-well plate in D10F medium. The cells were then infected for 2 h with *S*. Typhimurium (MOI 100) in serum-free DMEM to avoid interference from serum components, using the same treatment groups as those for adhesion and invasion assays. After infection, cells were detached by vigorous pipetting and collected by centrifugation at 8,000 rpm for 5 min. The collected supernatants were then processed according to the manufacturer’s instructions. Cytotoxicity was calculated based on absorbance at 490 nm and 680 nm using a SpectraMax M3 microplate reader (Molecular Devices, San Jose, CA, USA). To determine LDH activity, the 680 nm absorbance value (background) was subtracted from the 490 nm absorbance, and the results were calculated using the following formula:

$$\begin{array}{rl} & \mathrm{Cytotoxicity}\left(\%\right)\\ & \;\;\;\;\;\;\;\;\;\;\;\;\;\;\;\;\;\;\;\;\;=\left(\frac{OD_{\mathrm{treated}}-OD_{\mathrm{negative}}}{OD_{\mathrm{positive}}-OD_{\mathrm{negative}}}\right)\times 100 \end{array}$$

OD_treated_ : OD_490 nm_ - OD_680 nm_ of samples treated with *S*. Typhimurium and/or gut microbiota.

OD_positive_ : OD_490 nm_ - OD_680 nm_ of samples treated with the lysis buffer provided in the kit.

OD_negative_ : OD_490 nm_ - OD_680 nm_ of samples treated nothing.

### Western blot assay

Caco-2 cells were cultured in 6-well plates and infected with *S*. Typhimurium (MOI 100) for 2 h according to the invasion assay. After infection, the cells were washed twice with PBS and then lysed using lysis buffer (50 mM Tris-HCl, pH 8.0, 150 mM NaCl, 1 mM EDTA, 1 mM EGTA, and 1% NP-40) supplemented with a protease inhibitor cocktail (Quartett Biotechnologie GmbH, Potsdam, Germany). The protein concentration in the lysates was determined using the Bio-Rad DC Protein Assay kit (Bio-Rad Laboratories). The protein samples (40 μg per sample) were separated by SDS-PAGE and transferred to polyvinylidene difluoride membranes (EMD Millipore Co., Billerica, MA, USA). The membranes were blocked with 5% nonfat milk for 2 h at room temperature, followed by overnight incubation at 4°C with primary antibodies against IL-1β, IL-6, c-Jun, and GAPDH (Santa Cruz Biotechnology, Santa Cruz, CA, USA). The membranes were then incubated with goat anti-mouse IgG(H+L)-HRP secondary antibody (GenDEPOT) for 1 h at room temperature. Protein bands were visualized using EzWestLumi plus (ATTO Co., Tokyo, Japan) and the Invitrogen iBright-1500 imaging system (Thermo Fisher Scientific, Cleveland, OH, USA). Densitometric analysis of the bands was performed using FIJI ImageJ software (NIH, Bethesda, MA, USA), and the expression levels of IL-1β, IL-6, and c-Jun were normalized to GAPDH as an internal control reflecting the viable Caco-2 cell population.

### Statistical analysis

All statistical analyses and graphs were performed using GraphPad Prism version 10.2.1 (GraphPad Software, Boston, Massachusetts, USA) and IBM SPSS Statistics version 29 (IBM Corp., Armonk, NY, USA). Differences in alpha diversity among groups were assessed using two-tailed Mann–Whitney U tests with Bonferroni correction for multiple comparisons. Adjusted *p*-values < 0.05 were considered statistically significant. Beta diversity was assessed using Bray–Curtis dissimilarity and visualized by principal coordinate analysis (PCoA) in QIIME 2. Differences in adhesion, invasion, cytotoxicity, and densitometric analyses were analyzed by one-way ANOVA followed by Bonferroni-adjusted multiple-comparisons tests. Differences in *S*. Typhimurium growth and genus-level abundance across time points and treatment groups were analyzed by two-way ANOVA followed by Bonferroni-adjusted multiple-comparisons tests. All experiments were performed in triplicate for each sample and independently repeated three times.

## Results

### Growth of gut microbiota and S. Typhimurium during fecal fermentation

To investigate the dynamic changes of gut microbiota and *S*. Typhimurium during fecal fermentation, microbial growth was monitored. At 24 h, the concentration of gut microbiota continued to increase from 8.97 and 7.80 log GC/mL, reaching 10.43 and 10.81 log GC/mL in *S*. Typhimurium-infected and non-infected samples, respectively (Figure 1). In MiPro medium, *S*. Typhimurium increased from 7.36 to 8.28 log CFU/mL over 24 h, whereas in the presence of gut microbiota, its population decreased to 7.87 log CFU/mL at 24 h.

**Figure 1.**
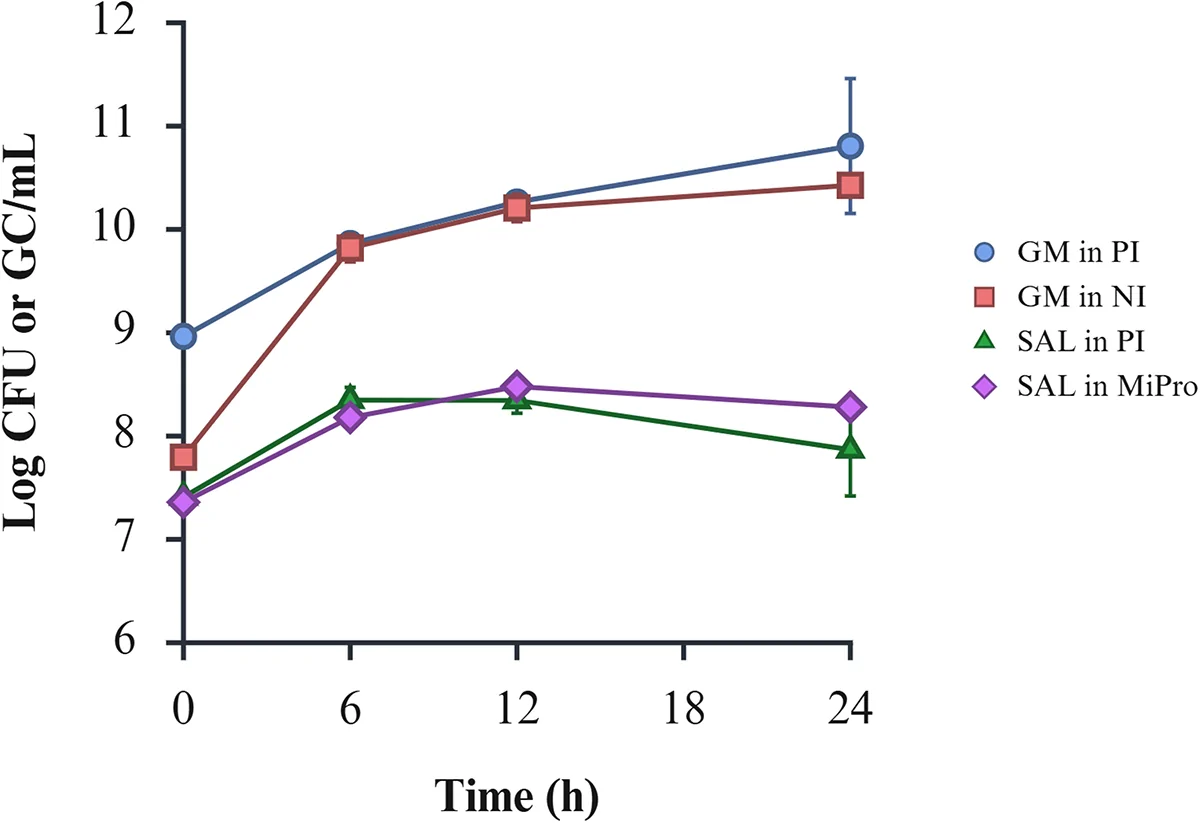
Bacterial concentration during incubation: post-infected gut microbiota (GM in PI; blue), non-infected gut microbiota (GM in NI; red), *S*. Typhimurium in the post-infected condition (SAL in PI; green), and the concentration of *S*. Typhimurium in MiPro medium (SAL in MiPro; violet) during incubation.

### Diversity and temporal dynamics of gut microbiota

Gut microbiota diversity was assessed based on incubation time and infection status. The Chao1 richness index decreased from 0 to 12 h (*p* = 0.024) and subsequently increased at 24 h (*p* = 0.782), whereas the Shannon diversity index decreased at 6 h (*p* = 0.024) and gradually increased at 24 h (*p* = 0.011) (Figure 2(A,B)). Both richness and evenness were reduced in *S*. Typhimurium-infected (PI) and non-infected (NI) groups compared to the control group (Figure 2(C,D)). Richness was lower and evenness was higher in the infected group than in the non-infected group, although these differences were not statistically significant. Beta diversity analysis revealed that samples at 6 h differed the most from those at 0 h, while samples at 12 h and 24 h were positioned closer to 0 h (Figure 2(E)). At 6 h, the infected and non-infected groups were clearly separated, and this difference was also observed at 12 h and 24 h.

**Figure 2.**
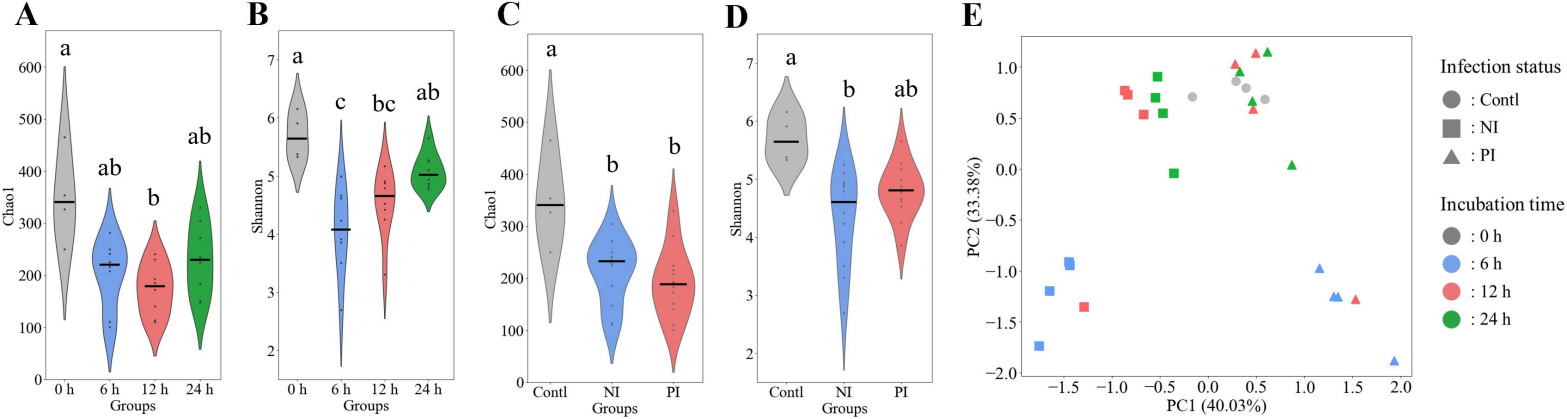
Violin plots and principal coordinate analysis (PCoA) comparing the alpha and beta diversity of gut microbiota during fecal fermentation according to incubation time (A, B) and infection status (C, D). Alpha diversity was assessed using the Chao1 richness index (A, C) and Shannon diversity index (B, D). Beta diversity was visualized by PCoA based on Bray–Curtis dissimilarity (E). Significant differences among groups are indicated by different lowercase letters (*p* < 0.05). Contl, control; NI, non-infected; PI, post-infected.

At 0 h, Bacillota (40.85%) and Bacteroidota (40.89%) were the dominant phyla, followed by Pseudomonadota (formerly Proteobacteria) at 7.14% of the total community (Figure S1). Over time, Bacillota declined markedly in both the infected and non-infected groups but partially recovered by 24 h. In contrast, Bacteroidota in the infected group and Pseudomonadota in the non-infected group increased at 6 h and gradually decreased toward 24 h.

### Compositional changes in gut microbiota by S. Typhimurium infection

*S*. Typhimurium infection did not affect the total bacterial load, as confirmed by qPCR (Figure 1), however, it resulted in a significant reduction in the abundance of Pseudomonadota (*p* < 0.001 at 6 h and 12 h, and *p* = 0.016 at 24 h) and a significant increase in Bacteroidota abundance (*p* < 0.001 at 6 h and *p* = 0.005 at 12 h). The abundance of Pseudomonadota ranged from 19.88% to 55.07% in the non-infected group, whereas it declined to 2.83–3.90% in the infected group (Figure 3(A)). Bacteroidota showed significantly higher abundance in the infected group compared to the non-infected group, increasing from 31.52% to 81.85% at 6 h and from 33.97% to 53.74% at 12 h (Figure 3(B)). At 24 h, the abundance of Bacteroidota was higher in the infected group (46.07%) than in the non-infected group (37.11%), with no significant differences. Bacillota abundance remained stable regardless of infection, ranging from 9.12% to 36.03% in the non-infected group and from 12.60% to 42.23% in the infected group (Figure 3(C)).

**Figure 3.**
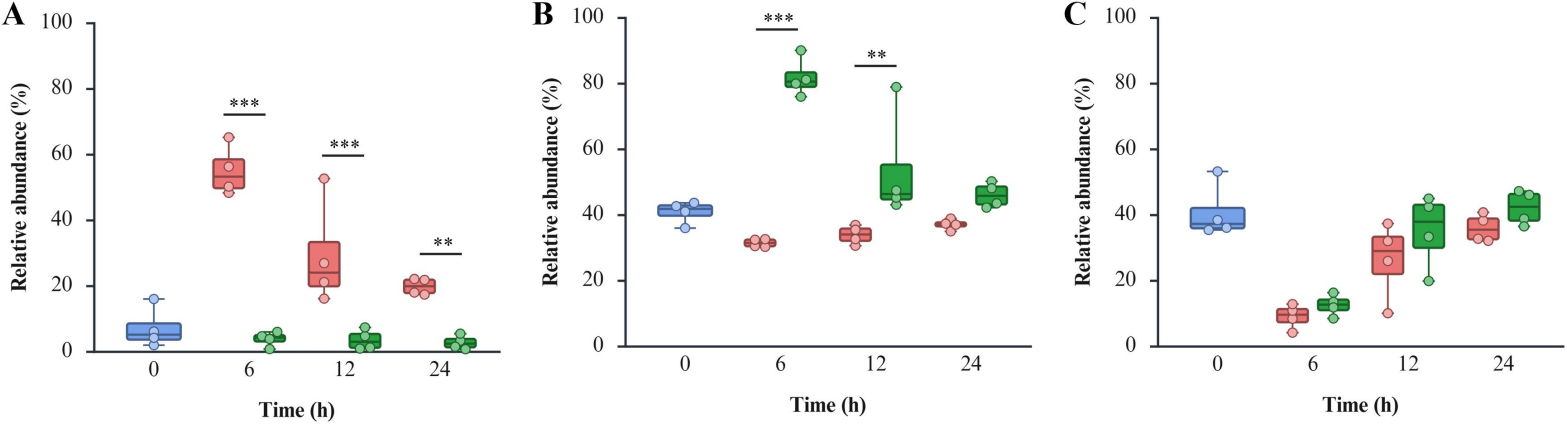
Relative abundance of three phyla of interest: (A) Pseudomonadota, (B) Bacteroidota, and (C) Bacillota. The color of each bar chart represents different infection conditions: control (blue), non-infected (red), and post-infected (green). Each dot represents the value for each sample. Statistically significant differences between the non-infected and post-infected groups are indicated by asterisks (*p < 0.05; **p < 0.01; ***p < 0.001).

The relative abundance of *Escherichia-Shigella* was 17.25–52.98% in the non-infected group and reduced to 0.91–2.92% in the infected group during incubation (*p* < 0.001 at 6 h and 12 h, and *p* = 0.024 at 24 h) (Figure 4(A)). In contrast, several SCFA-associated genera, including *Bacteroides*, *Parabacteroides*, *Lachnoclostridium*, *Clostridium*_*sensu*_*stricto*_1, and *Hungatella*, showed higher relative abundances in the infected group. *Bacteroides* and *Parabacteroides* exhibited relative abundances of 39.60–73.15% and 5.60–8.22% in the infected group, respectively, compared with 28.53–32.03% and 2.83–4.69% in the non-infected group (Figure 4(B,C)). The relative abundances of *Lachnoclostridium*, *Clostridium*_*sensu*_*stricto*_*1*, and *Hungatella* ranged from 0.32–6.52%, 0.54–2.58%, and 0.19–1.08% in the non-infected group, respectively, compared with 0.76–8.42%, 1.98–5.31%, and 0.54–1.74% in the infected group (Figure 4(D–F)).

**Figure 4.**
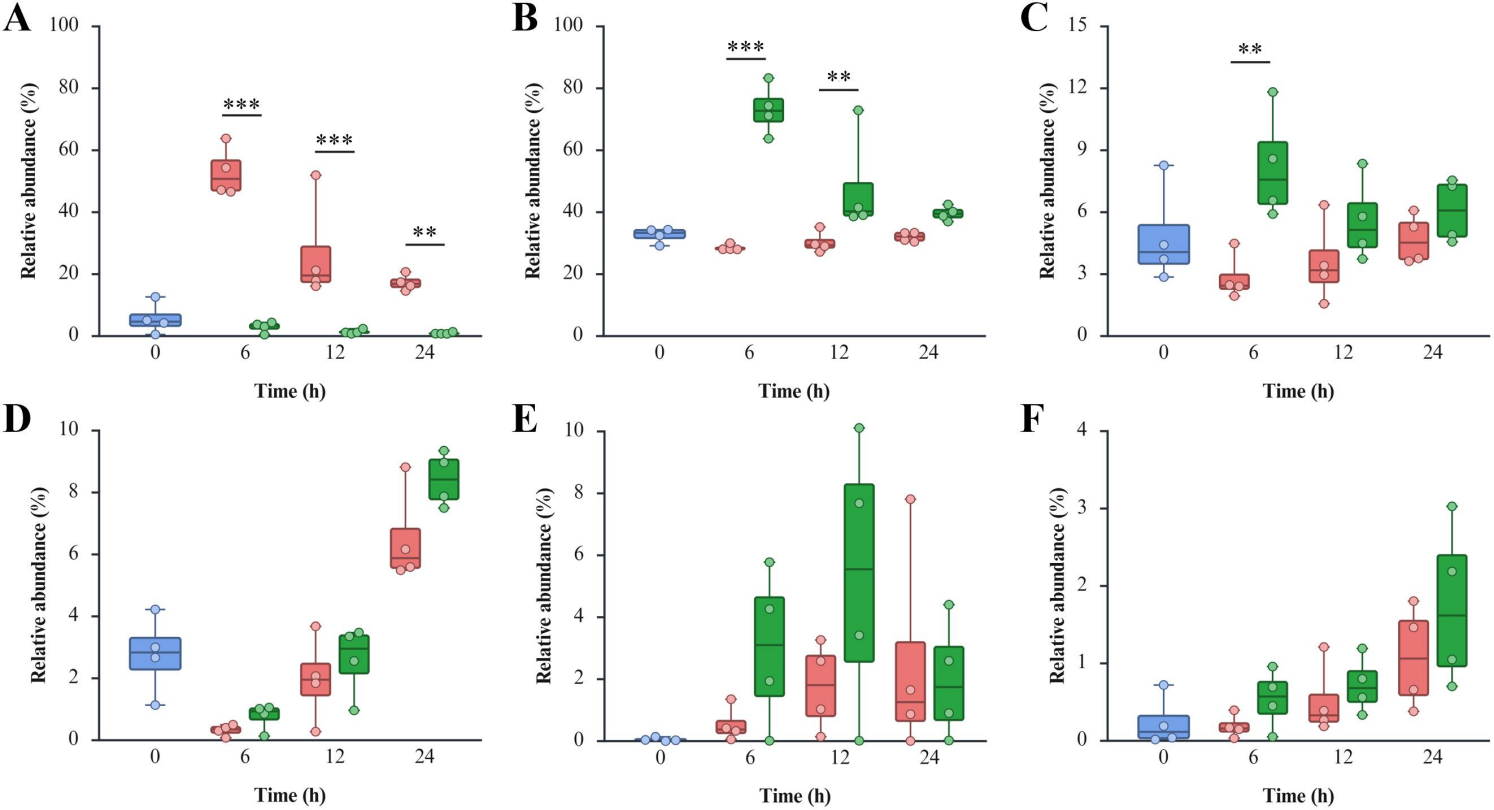
Relative abundance of six genera of interest: (A) *Escherichia-Shigella*, (B) *Bacteroides*, (C) *Parabacteroides*, (D) *Lachnoclostridium*, (E) *Clostridium*_*sensu*_*stricto*_*1*, and (F) *Hungatella*. The color of each bar chart represents different infection conditions: control (blue), non-infected (red), and post-infected (green). Each dot represents the value for each sample. Statistically significant differences between the non-infected and post-infected groups are indicated by asterisks (*p < 0.05; **p < 0.01; ***p < 0.001).

### Changes in SCFAs and ethanol

SCFA and ethanol concentrations showed no notable differences between infected and non-infected groups during incubation (Figure 5). Acetate levels ranged from 0 to 1.63 mg/mL in the non-infected group and 0 to 1.55 mg/mL in the infected group, and propionate levels varied from 0 to 1.15 mg/mL and 0 to 1.18 mg/mL, respectively. Ethanol levels were also comparable, measuring up to 0.95 mg/mL in the non-infected group and 0.94 mg/mL in the infected group. While butyrate was not detected at 12 h in the infected group, it was detected at 24 h (0.95 mg/mL), with concentrations lower than those in the non-infected group (1.11 mg/mL).

**Figure 5.**
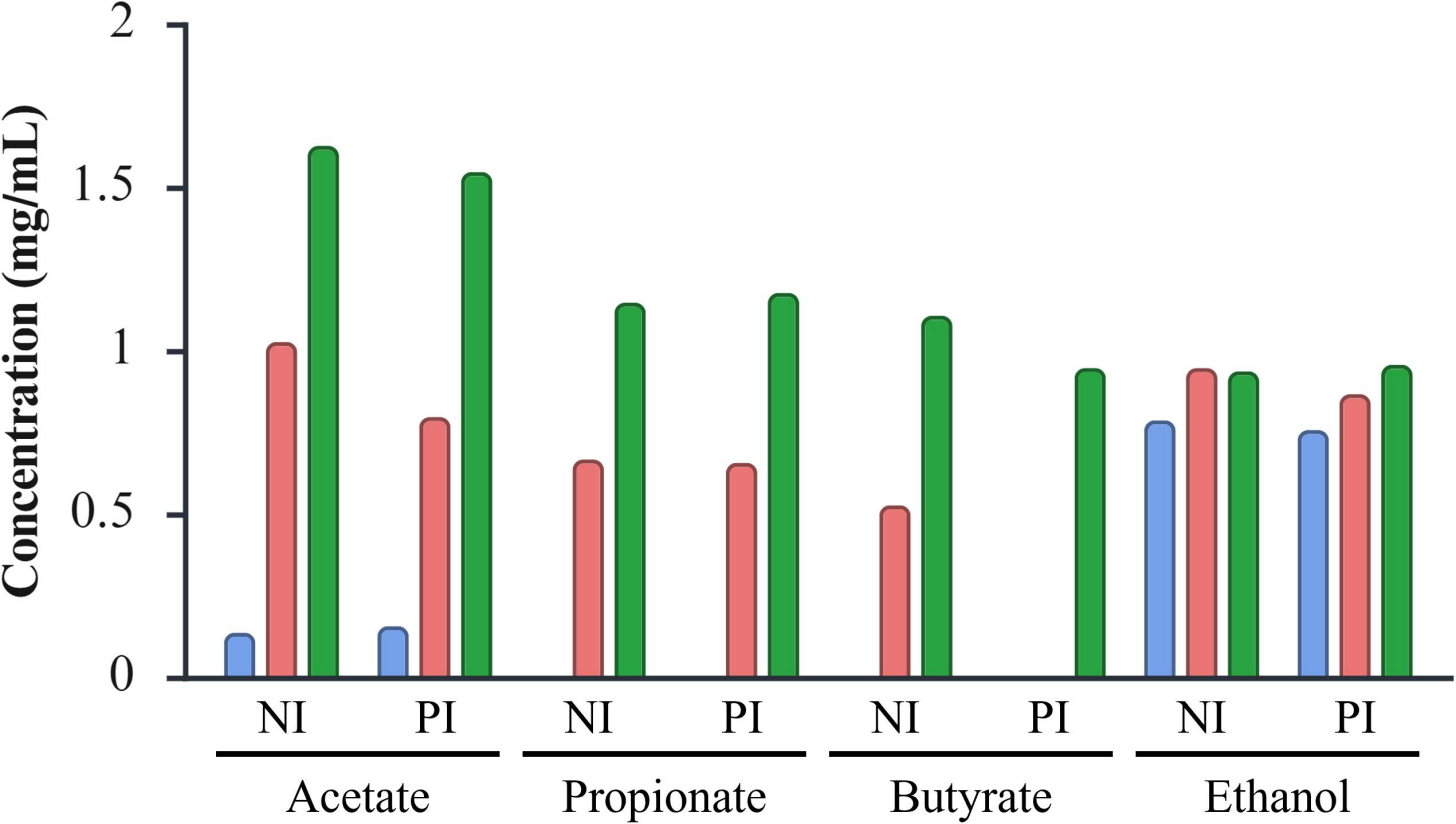
The concentration of short-chain fatty acids and ethanol of *S*. Typhimurium-infected and non-infected pooled fecal samples during fermentation. The color of each bar chart represents the different incubation times: 6 h (blue), 12 h (red), and 24 h (green). NI, non-infected; PI, post-infected.

### Adhesion, invasion, and cytotoxicity in caco-2 cells

Co-treatment with gut microbiota inhibited the adhesion and invasion of *S*. Typhimurium in Caco-2 cells and reduced cytotoxicity, with the protective effect increasing in a dose-dependent manner. *S*. Typhimurium showed an adhesion level of 6.75 log CFU/mL to Caco-2 cells and an invasion level of 5.67 log CFU/mL (Figure 6(A,B)). Adhesion was significantly reduced to 76.38% (*p* < 0.001) in the GtoS ratio 100:1 group and 86.57% (*p* = 0.005) in the GtoS 10:1 group compared to the control. Invasion levels were also significantly lower in the GtoS 100:1 group (66.11%, *p* = 0.002) and the GtoS 10:1 group (79.15%, *p* = 0.041) compared to the control. Among the gut microbiota-treated (GM) groups, cytotoxicity was the lowest in the GM 9 log group (−14.40%), followed by the GM 8 log group (−1.10%) and GM 7 log group (5.49%) (Figure 6(C)). Cytotoxicity induced by *S*. Typhimurium infection was 44.10%, but was significantly reduced to −5.59% in the GtoS 100:1 group (*p* = 0.004), −0.97% in the GtoS 10:1 group (*p* = 0.009), and 5.84% in the GtoS 1:1 group (*p* = 0.033).

**Figure 6.**
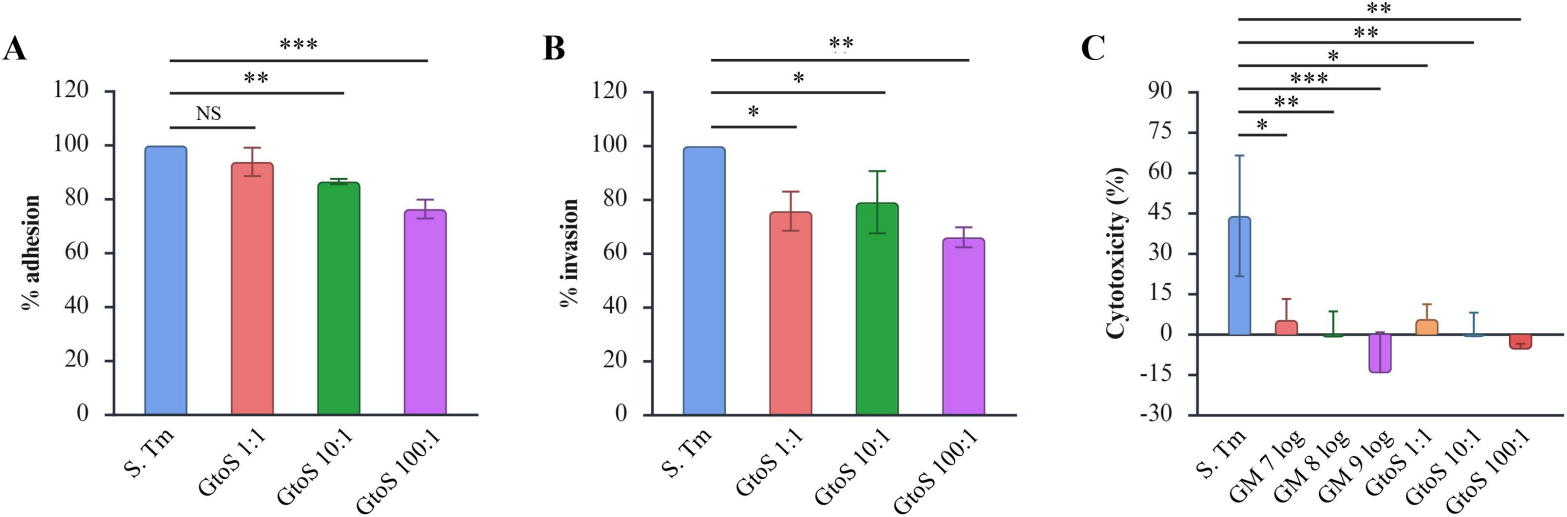
Inhibitory effects of gut microbiota on *S*. Typhimurium infection. (A) Adhesion and (B) Invasion of *S*. Typhimurium (7 log CFU/mL) to Caco-2 cells in the presence of gut microbiota at various gut microbiota-to-*S*. Typhimurium (GtoS: 1:1, 10:1, and 100:1). (C) Cytotoxicity of *S*. Typhimurium and gut microbiota at different concentrations (7, 8, and 9 log CFU/mL), as well as in co-culture at different GtoSs (1:1, 10:1, and 100:1). Bars represent mean ± sd from three independent experiments. Significant differences compared to the *S*. Typhimurium control are indicated by asterisks (*p < 0.05; **p < 0.01; ***p < 0.001). S.Tm, *S*. Typhimurium; GM, gut microbiota; NS, non-significant.

### Inflammatory responses in caco-2 cells

The expressions of IL-1β, IL-6, and c-Jun were progressively inhibited by increasing concentrations of gut microbiota. *S*. Typhimurium infection significantly upregulated these inflammatory markers, with band intensities reaching 677.07% for IL-6, 138.12% for IL-1β, and 109.42% for c-Jun relative to the control (Figure 7). However, co-treatment with gut microbiota suppressed this increase in a dose-dependent manner. The expression levels of IL-1β, IL-6, and c-Jun were significantly reduced to 31.73%, 21.79%, and 44.47% in the GtoS 1:1 group; 6.81%, 4.48%, and 44.47% in the GtoS 10:1 group; and 3.29%, 2.28%, and 4.01% in the GtoS 100:1 group, respectively (*p* < 0.001). No significant differences were observed between the GtoS 1:1 and GtoS 10:1 group for any inflammatory markers.

**Figure 7.**
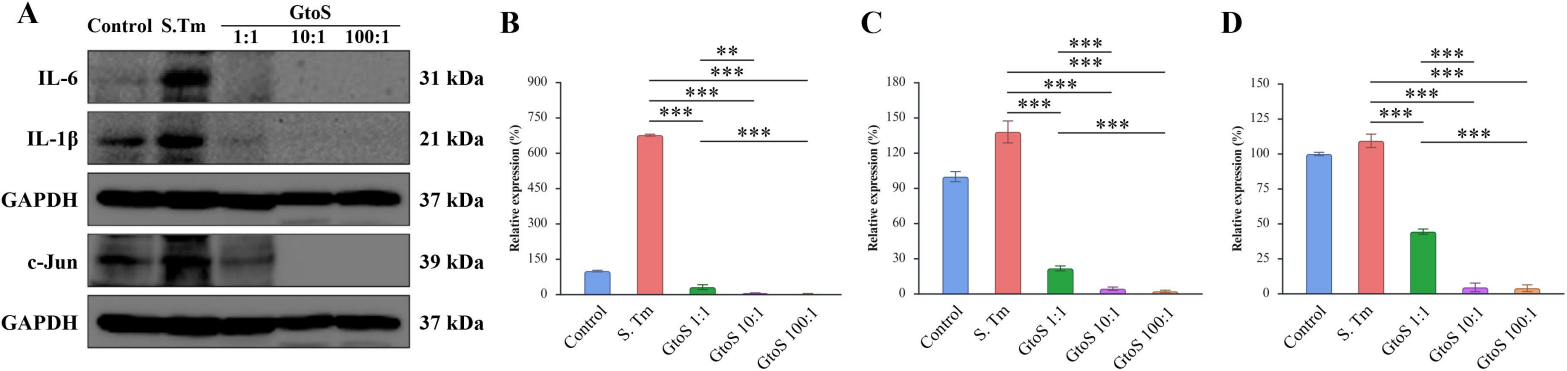
Gut microbiota attenuates *S*. Typhimurium-induced inflammatory responses in Caco-2 cells. (A) Western blot analysis of IL-6, IL-1β, and c-Jun expression after infection with *S*. Typhimurium (7 log CFU/mL) alone or co-treated with gut microbiota at different gut microbiota-to-*S*. Typhimurium (GtoS: 1:1, 10:1, and 100:1). GAPDH was used as a loading control. (B-D) Densitometric quantification of IL-6 (B), IL-1β (C), and c-Jun (D) protein expression levels, shown as percentage relative to the control group (set to 100%). Bars represent mean ± sd from three independent experiments. Statistical differences between two groups are indicated by asterisks (**p < 0.01; ***p < 0.001). *S*. Tm, *S*. Typhimurium alone; GM, gut microbiota.

## Discussion

*S*. Typhimurium is a major pathogenic bacterium that disrupts gut microbial homeostasis and induces intestinal inflammation [2]. After reaching the large intestine, *S*. Typhimurium adheres to and invades the intestinal epithelium, initiating infection [2]. The human gastrointestinal tract harbors approximately 10^12^-10^14^ CFU/mL of gut microbiota, which engages in bidirectional interactions with *S*. Typhimurium during its colonization of the gut [2,7]. *S*. Typhimurium can disrupt gut microbial homeostasis, altering the abundance of commensal bacteria, and exploiting nutrient competition to promote its survival [2]. Conversely, gut microbiota serves as a crucial defense barrier, limiting *S*. Typhimurium colonization through competitive exclusion, production of antimicrobial metabolites, and modulation of the host immune response [2,3]. In this study, we investigated the interaction between gut microbiota and *S*. Typhimurium, focusing on changes in the microbial composition and metabolite profiles, as well as alterations in *S*. Typhimurium adhesion, invasion, and host inflammatory responses.

Gut microbiota maintains intestinal homeostasis through habitat filtering and microbial competition but can undergo transient dysbiosis following perturbations such as antibiotic treatment, immune responses, or pathogen infection [2,21]. Despite such disturbances, the microbiota often exhibits resilience by gradually returning toward its pre-disturbance state [21]. In the present study, substantial changes in microbial diversity and community structure were observed during fecal fermentation. The decline in Bacillota abundance and microbial diversity may partly reflect oxygen exposure during fecal sampling and handling, which can compromise the survival of strict anaerobes under partially aerobic *in vitro* conditions [2]. The partial recovery of community evenness and Bacillota abundance over time suggests gradual stabilization following the initial disturbance. Although gut microbiota resilience is influenced by host immunity, the intestinal environment, and diet, the present *in vitro* findings suggest that interbacterial interactions may also contribute to community stabilization [21].

Pseudomonadota, particularly *Escherichia-Shigella*, was more abundant in non-infected samples, whereas this increase was attenuated in *Salmonella*-infected samples. Because *Escherichia-Shigella* and *Salmonella* both belong to the *Enterobacteriaceae*, this pattern may reflect competitive or inhibitory interactions within this bacterial family. Although *Salmonella*-associated expansion of *Enterobacteriaceae* has been linked to host-derived changes such as increased oxygen and lactate availability [9,22], these host-mediated mechanisms were absent in the present *in vitro* model, suggesting that bacteria–bacteria interactions may have contributed to the observed response. One possible explanation is that the reduction in *Escherichia-Shigella* may result from type VI secretion system-mediated antagonism or salmochelin-mediated competition for iron, both of which could provide *S*. Typhimurium with a competitive advantage over neighboring Gram-negative bacteria [2].

SCFA-producing bacteria, particularly *Bacteroides*, were also increased markedly in the infected groups. The mechanism by which *S*. Typhimurium may promote the increase in SCFA-producing bacteria remains unclear, as limited evidence is currently available to explain this interaction. Nevertheless, previous studies suggest that increased abundance of SCFA-producing bacteria may contribute to the suppression of *Salmonella*. Administration of *B. thetaiotaomicron* to mice harboring gut microbiota with low Bacteroidota abundance enhanced colonization resistance against *Salmonella*, whereas this effect was absent in a propionate-deficient mutant [4]. Acetate and propionate inhibited the growth and virulence of enteric pathogens by lowering intestinal pH and interfering with bacterial metabolism [2]. However, quantification of fermentation metabolites revealed that overall SCFA and ethanol levels were broadly comparable between the infected and non-infected groups, although butyrate showed a tendency to decrease in the infected group at later time points. Therefore, the present study cannot conclusively determine whether the observed suppression of *S*. Typhimurium was directly attributable to SCFA-mediated effects.

Gut microbiota significantly inhibited the adhesion and invasion of *S*. Typhimurium and reduced *S*. Typhimurium-induced cytotoxicity in Caco-2 cells. These protective effects may involve direct mechanisms such as nutrient competition, antimicrobial compound production, and niche exclusion [10], thereby contributing to the maintenance of epithelial barrier integrity [2]. Notably, gut microbiota treatment alone also reduced cytotoxicity compared with the untreated control, suggesting that the microbiota may support epithelial cell stability and viability even in the absence of pathogen challenge [3]. Further studies using cell-free supernatants from fecal fermentation would be valuable for determining the specific contribution of microbiota-derived metabolites to the attenuation of *S*. Typhimurium virulence and host cell responses.

Toll-like receptors and NOD-like receptors on epithelial cells recognize *Salmonella* pathogen-associated molecular patterns, such as lipopolysaccharide and flagellin, thereby activating NF-κB and MAPK signaling pathways [23,24]. These pathways promote the transcription of IL-6 and pro-IL-1β, and activate c-Jun, leading to the expression of inflammation-related genes. In the present study, *S*. Typhimurium infection significantly increased IL-1β, IL-6, and c-Jun expression, whereas co-treatment with gut microbiota attenuated these responses in a dose-dependent manner, consistent with the observed reduction in cytotoxicity. These findings suggest that gut microbiota may protect epithelial cells not only by limiting pathogen-associated damage but also by suppressing *S*. Typhimurium-induced inflammatory signaling. Although the underlying mechanisms cannot be fully resolved in the present *in vitro* model, the differential responses of Caco-2 cells to gut microbiota and *S*. Typhimurium suggest that epithelial innate immune signaling remains responsive to different types of bacterial exposure.

## Conclusion

This study provides evidence that gut microbiota can contribute to resistance against *S*. Typhimurium through microbiota–pathogen interactions and modulation of pathogen-induced host responses. Gut microbiota modulated *S*. Typhimurium-induced changes in microbial community structure and may contribute to colonization resistance through direct bacteria–bacteria interactions. In Caco-2 cells, gut microbiota also reduced *S*. Typhimurium adhesion, invasion, cytotoxicity, and inflammatory responses, indicating a protective effect against pathogen-induced epithelial damage. Although the *in vitro* models do not fully reproduce the complexity of the intestinal environment, including host-derived factors, mucus, spatial organization, and oxygen gradients, they provide a useful framework for investigating microbiota–pathogen interactions and their effects on intestinal epithelial responses. Further studies incorporating host and immune components will be necessary to clarify the underlying mechanisms and validate their relevance *in vivo*.

## Supplementary Material

Supplementary information.docx

## Data Availability

All datasets supporting the findings of this study are publicly available under an open license. The complete processed datasets used for statistical analyses, including the values underlying all means, standard deviations, graphs, and figures, as well as the numerical data extracted from images, have been deposited in Zenodo [25] under a CC-BY 4.0 license at https://doi.org/10.5281/zenodo.18465955. Raw 16S rRNA gene amplicon sequencing data generated in this study have been deposited in the NCBI Sequence Read Archive (SRA) under BioProject accession number PRJNA1141279: https://www.ncbi.nlm.nih.gov/sra/PRJNA1141279. These datasets allow full replication of all analyses and figures reported in this manuscript. No data are subject to embargo or access restrictions.

## References

[1] Zheng D, Liwinski T, Elinav E. Interaction between microbiota and immunity in health and disease. Cell Res. 2020;30(6):492–11. doi: 10.1038/s41422-020-0332-7 PMC7264227 32433595

[2] Rogers AWL, Tsolis RM, Bäumler AJ. *Salmonella* versus the microbiome. Microbiol Mol Biol Rev. 2021;85(1):e00027–19. doi: 10.1128/mmbr.00027-19 PMC8549850 33361269

[3] Singh S, Koo OK. A comprehensive review exploring the protective role of specific commensal gut bacteria against *salmonella*. Pathogens. 2024;13(8):642. doi: 10.3390/pathogens13080642 PMC11356920 39204243

[4] Jacobson A, Lam L, Rajendram M, et al. A gut commensal-produced metabolite mediates colonization resistance to *salmonella* infection. Cell Host Microbe. 2018;24(2):296–307.e7. doi: 10.1016/j.chom.2018.07.002 PMC6223613 30057174

[5] Peng L, Li ZR, Green RS, et al. Butyrate enhances the intestinal barrier by facilitating tight junction assembly via activation of AMP-activated protein kinase in caco-2 cell monolayers. J Nutr. 2009;139(9):1619–1625. doi: 10.3945/jn.109.104638 PMC2728689 19625695

[6] Kirk MD, Pires SM, Black RE, et al. World Health Organization estimates of the global and regional disease burden of 22 foodborne bacterial, protozoal, and viral diseases, 2010: a data synthesis. PLoS Med. 2015;12(12):e1001921. doi: 10.1371/journal.pmed.1001921 PMC4668831 26633831

[7] Aljahdali NH, Sanad YM, Han J, et al. Current knowledge and perspectives of potential impacts of *Salmonella enterica* on the profile of the gut microbiota. BMC Microbiol. 2020;20(1):353. doi: 10.1186/s12866-020-02008-x PMC7673091 33203384

[8] Shin NR, Whon TW, Bae JW. Proteobacteria: microbial signature of dysbiosis in gut microbiota. Trends Biotechnol. 2015;33(9):496–503. doi: 10.1016/j.tibtech.2015.06.011 26210164

[9] Velazquez EM, Nguyen H, Heasley KT, et al. Endogenous *Enterobacteriaceae* underlie variation in susceptibility to *salmonella* infection. Nat Microbiol. 2019;4(6):1057–1064. doi: 10.1038/s41564-019-0407-8 PMC6533147 30911125

[10] Deng L, Wang S. Colonization resistance: the role of gut microbiota in preventing *salmonella* invasion and infection. Gut Microbes. 2024;16(1):2424914. doi: 10.1080/19490976.2024.2424914 PMC11552263 39514544

[11] Palmer AD, Slauch JM. Mechanisms of *salmonella* pathogenesis in animal models. Hum Ecol Risk Assess. 2017;23(8):1877–1892. doi: 10.1080/10807039.2017.1353903 PMC6484827 31031557

[12] Krych L, Hansen CH, Hansen AK, et al. Quantitatively different, yet qualitatively alike: a meta-analysis of the mouse core gut microbiome with a view towards the human gut microbiome. PLOS ONE. 2013;8(5):e62578. doi: 10.1371/journal.pone.0062578 PMC3641060 23658749

[13] Stecher B, Chaffron S, Käppeli R, et al. Like will to like: abundances of closely related species can predict susceptibility to intestinal colonization by pathogenic and commensal bacteria. PLoS Pathog. 2010;6(1):e1000711. doi: 10.1371/journal.ppat.1000711 PMC2796170 20062525

[14] Wos-Oxley ML, Bleich A, Oxley AP, et al. Comparative evaluation of establishing a human gut microbial community within rodent models. Gut Microbes. 2012;3(3):234–249. doi: 10.4161/gmic.19934 PMC3427216 22572831

[15] Li L, Abou-Samra E, Ning Z, et al. An *in vitro* model maintaining taxon-specific functional activities of the gut microbiome. Nat Commun. 2019;10(1):4146. doi: 10.1038/s41467-019-12087-8 PMC6742639 31515476

[16] Kim DW, Singh S, Kim UI, et al. Exploring the fate of *Listeria monocytogenes* in an *in vitro* digestion and fecal fermentation model: insights into survival during digestion and interaction with gut microbiota. Front Microbiol. 2025;16:1616720. doi: 10.3389/fmicb.2025.1616720 PMC12325261 40771694

[17] Swanson KS, Gibson GR, Hutkins R, et al. The International Scientific association for Probiotics and Prebiotics (ISAPP) consensus statement on the definition and scope of synbiotics. Nat Rev Gastroenterol Hepatol. 2020;17(11):687–701. doi: 10.1038/s41575-020-0344-2 PMC7581511 32826966

[18] Fuller Z, Louis P, Mihajlovski A, et al. Influence of cabbage processing methods and prebiotic manipulation of colonic microflora on glucosinolate breakdown in man. Br J Nutr. 2007;98(2):364–372. doi: 10.1017/S0007114507709091 17403273

[19] Jankowska A, Laubitz D, Antushevich H, et al. Competition of *Lactobacillus Paracasei* with *Salmonella enterica* for adhesion to caco-2 cells. J Biomed Biotechnol. 2008;2008(1):357964. doi: 10.1155/2008/357964 PMC2279207 18401442

[20] Maldonado Y, Fiser JC, Nakatsu CH, et al. Cytotoxicity potential and genotypic characterization of *Escherichia coli* isolates from environmental and food sources. Appl Environ Microbiol. 2005;71(4):1890–1898. doi: 10.1128/AEM.71.4.1890-1898.2005 PMC1082550 15812017

[21] Lozupone CA, Stombaugh JI, Gordon JI, et al. Diversity, stability and resilience of the human gut microbiota. Nature. 2012;489(7415):220–230. doi: 10.1038/nature11550 PMC3577372 22972295

[22] Khan S, Chousalkar KK. *Salmonella* Typhimurium infection disrupts but continuous feeding of *Bacillus* based probiotic restores gut microbiota in infected hens. J Anim Sci Biotechnol. 2020;11(1):29. doi: 10.1186/s40104-020-0433-7 PMC7087389 32211190

[23] Tallant T, Deb A, Kar N, et al. Flagellin acting via TLR5 is the major activator of key signaling pathways leading to NF-κB and proinflammatory gene program activation in intestinal epithelial cells. BMC Microbiol. 2004;4(1):33. doi: 10.1186/1471-2180-4-33 PMC516440 15324458

[24] Yang F, Sheng X, Huang X, et al. Interactions between *Salmonella* and host macrophages – dissecting NF-κB signaling pathway responses. Microb Pathog. 2021;154:104846. doi: 10.1016/j.micpath.2021.104846 33711426

[25] Kim DW, Singh S, Je HJ, et al. *In vitro* study of gut microbiota-mediated response to *Salmonella* Typhimurium with a focus on dynamics and immune modulation [dataset]. Zenodo. 2026. doi: 10.5281/zenodo.18465955 PMC13757655 42989820

