# Supplementary material for: In vitro study of gut microbiota-mediated response to Salmonella Typhimurium with a focus on dynamics and immune modulation
Source: Virulence. 2026 Nov 5;17(1):2758464. doi: 10.1080/21505594.2026.2758464 (PMC13757655; doi:10.1080/21505594.2026.2758464)
Supplement: Supplementary file 1 [file KVIR_A_2758464_SM3791.docx]

*Supplementary Information*

Figure S1. Relative abundance of gut microbiota at the phylum level during fecal fermentation. PF, pooled fecal samples; IFS, individual fecal samples; Contl, control; NI, non-infected; PI, post-infected.
